# A call to look beyond prescription opioid supply-side restrictions and include health equity when predicting opioid policy effectiveness

**DOI:** 10.1016/j.lana.2021.100075

**Published:** 2021-09-10

**Authors:** Amie Goodin, Juan M. Hincapie-Castillo

**Affiliations:** 1 Pharmaceutical Outcomes and Policy, University of Florida, USA; 2 Center for Drug Evaluation and Safety, University of Florida, USA; 3 Epidemiology, University of North Carolina Chapel Hill, USA; 4 Injury Prevention Research Center, University of North Carolina Chapel Hill, USA

We read with interest the study by Rao and colleagues on opioid policy effectiveness, which extends their previous modeling efforts to predict opioid-related overdose, life-years, and QALYs [1,2]. This work presents a useful framework from which to investigate policy effectiveness.

We request further consideration, however, regarding the model inputs and underlying assumptions. Of most concern is the continued dominance of opioid prescription supply restriction policies as a means to *decrease* opioid-related harms. United States fatal and non-fatal opioid-involved overdose trends are evidence that opioid-involved mortality is no longer primarily associated with prescription opioids, but more often non-medical fentanyl analogues [3]. The authors concede this but do not adjust the policy portfolio included within the modeling exercise. Emerging evaluations, meanwhile, demonstrate that prescription restriction policies are associated with patient harms such as reduced access to pain treatments and increases in non-medical opioid-involved harms, including mortality [4].

The inclusion of policies within the model with stronger supporting evidence from public health intervention assessments for reducing opioid-related harms (e.g., naloxone availability, syringe exchange) were, not surprisingly, the policies most likely to be found as “uniformly beneficial” across examined outcomes. It is then unclear why the authors’ concluding statements endorsed policy options to reduce opioid prescribing rather than harm reducing policies. Lastly, the authors do not address the important health disparities and equity issues in both access to opioid medications for pain treatment and access to harm reduction strategies [5]. Thus, we encourage caution with the assumption of uniform policy effects without account for jurisdiction or patient characteristics.

## Declaration of competing interest

None.

## References

[1] Rao IJ, Humphreys K, Brandeau ML. (2021). Effectiveness of Policies for Addressing the US Opioid Epidemic: A Model-Based Analysis from the Stanford-Lancet Commission on the North American Opioid Crisis. Lancet Regional Health-Americas.

[2] Pitt AL, Humphreys K, Brandeau ML. (2018). Modeling Health Benefits and Harms of Public Policy Responses to the US Opioid Epidemic. American journal of public health.

[3] Mattson C, Tanz L, Quinn K, Kariisa M, Patel P, Davis N. (2021). Trends and Geographic Patterns in Drug and Synthetic Opioid Overdose Deaths - United States, 2013-2019. MMWR. Morbidity and mortality weekly report.

[4] Nicholson KM, Hellman D. (2021). Opioid Prescribing and the Ethical Duty to Do No Harm. American Journal of Law & Medicine.

[5] Morden N, Chyn D, Wood A, Meara E (2021). Racial Inequality in Prescription Opioid Receipt - Role of Individual Health Systems. The New England journal of medicine.

